# Supplementary material for: A whole-animal phenotypic drug screen identifies suppressors of atherogenic lipoproteins
Source: eLife. 2026 Aug 27;14:RP105314. doi: 10.7554/eLife.105314 (PMC13521566; doi:10.7554/eLife.105314)
Supplement: Supplementary file 2 [file elife-105314-code1.zip › JHDL_primary_result/Bismuth (III) oxychloride/Bismuth (III) oxychloride_JHDL_28_026_016_2019_12_06_11_27.pdf]

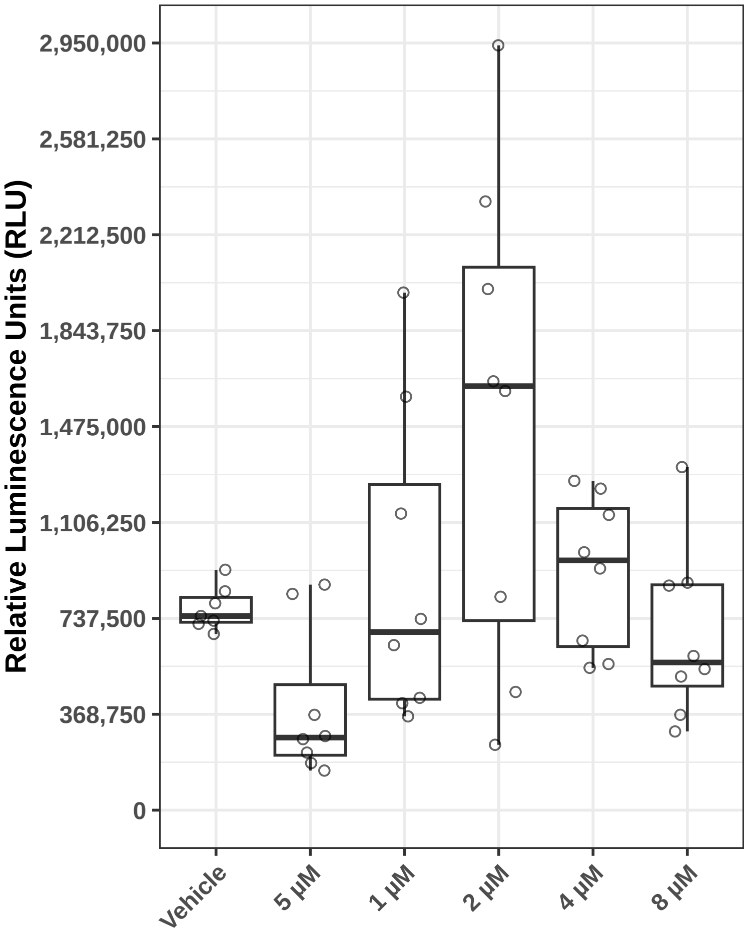
Relative Luminescence Units (RLU)
2,950,000
2,581,250
2,212,500
1,843,750
1,475,000
1,106,250
737,500
368,750
0
Vehicle
5 μM
1 μM
2 μM
4 μM
8 μM
